# A multi‑country evaluation of healthcare professionals’ experiences with the PanCareFollowUp training package to implement person-centred care in childhood cancer survivorship care: A PanCareFollowUp study

**DOI:** 10.1007/s00520-026-10930-5

**Published:** 2026-06-30

**Authors:** Jacqueline J. Loonen, Eline Bouwman, Adriaan Penson, Cecilia Follin, Anne Uyttebroeck, Dionne Breij, Katerina Kepakova, Sofie Prikken, Lars Hjorth, Jeanette F. Winther, Helena J. H. van der Pal, Carina Schneider, Anita Kienesberger, Hannah Gsell, Gisela Michel, Saskia M. F. Pluijm, Roderick Skinner, Leontien C. M. Kremer, Monica Muraca, Tomas Kepak, Rosella P. M. G. Hermens

**Affiliations:** 1 Center of Expertise for Cancer Survivorship, Radboud University Medical Center, Nijmegen, the Netherlands; 2 Society for Personalized Healthcare, Nijmegen, the Netherlands; 3 IQ Health, Radboud University Medical Center, Nijmegen, the Netherlands; 4 Department of Clinical Sciences Lund, Oncology, Lund University, Skåne University Hospital, Lund, Sweden; 5 Department of Pediatric Hematology and Oncology, University Hospitals Leuven, Leuven, Belgium; 6 Department of Oncology, Pediatric Oncology, KU Leuven, Leuven, Belgium; 7 International Clinical Research Center (FNUSA-ICRC), St. Anne’s University Hospital, Masaryk University, Brno, Czech Republic; 8 Department of Clinical Sciences Lund, Pediatrics, Lund University, Skåne University Hospital, Lund, Sweden; 9 Childhood Cancer Research Group, Danish Cancer Society Research Center, Copenhagen, Denmark; 10 Department of Clinical Medicine, Faculty of Health, Aarhus University and Aarhus University Hospital, Aarhus, Denmark; 11 Princess Máxima Center for Pediatric Oncology, Utrecht, the Netherlands; 12 Childhood Cancer International - Europe, Vienna, Austria; 13 Faculty of Health Sciences and Medicine, University of Lucerne, Lucerne, Switzerland; 14 Great North Children’s Hospital, Newcastle upon Tyne, UK; 15 Translational and Clinical Research Institute, Wolfson Childhood Cancer Research Center, Newcastle upon Tyne, UK; 16 Department of Pediatrics, Emma Children’s Hospital, Amsterdam UMC, Amsterdam, the Netherlands; 17 Faculty of Medicine, Utrecht University and Utrecht Medical Centre, Utrecht, the Netherlands; 18 Division of Pediatric Hematology and Oncology, IRCCS Istituto Giannina Gaslini, DOPO Clinic, Genoa, Italy

**Keywords:** Person-Centred Care, Cancer Survivorship Care, PanCareFollowUp, Implementation

## Abstract

**Purpose:**

Survivors of childhood, adolescent, and young adult (CAYA) cancer face lifelong risks of treatment-related late effects, emphasizing the need for innovative, person-centred survivorship care. The PanCareFollowUp (PCFU) Care intervention, based on person-centred care (PCC), prioritizes survivors' values and needs. This paper describes the evaluation of experiences of healthcare professionals’ (HCPs) and supporting staff’s experiences with the training package that was developed for implementation of PCC in the PCFU Care.

**Methods:**

To support PCC delivery, a training package, including a training presentation with voice-over and a workshop, was developed. Healthcare professionals (HCPs) and supporting staff from four European survivorship clinics evaluated the training package through questionnaires measuring readiness to apply PCC, knowledge, confidence, perceived utility, and tool-related experiences.

**Results:**

Eighteen HCPs evaluated the training presentation. Readiness to apply PCC increased from 68% pre-training to 89% post-training, while confidence declined from 78% to 68%. Most participants reported improved knowledge, with 94% indicating at least some increase. Frameworks such as Ekman’s pillars, the seven steps for a PCC consultation, and shared decision-making questions were rated highly useful. Twenty-eight HCPs evaluated the workshop; 82% found it useful and 82% reported an overall favourable impression. Participants valued open discussion, multidisciplinary perspectives, and survivor involvement. The workshop’s short duration and more broadly, limited time, staff shortages, and digital infrastructure were identified as barriers to PCC implementation.

**Conclusion:**

This multi-country evaluation shows that even brief PCC training can enhance HCPs’ readiness to deliver person-centred survivorship care. The findings highlight both the potential of concise educational interventions and the importance of delivery format and organizational context for the sustainable implementation of PCC across survivorship care settings.

**Supplementary Information:**

The online version contains supplementary material available at https://doi.org/10.1007/s00520-026-10930-5.

## Introduction

The population of childhood, adolescent and young adult (CAYA) cancer survivors is rapidly increasing with currently around 500,000 CAYA cancer survivors living in Europe [1, 2]. While advances in cancer treatment have markedly improved survival rates, they have also led to a substantial burden of long-term health complications, known as late effects, which pose lifelong risks for many survivors [3, 4]. To address these late effects, cancer survivorship care aims to prevent, detect and treat late effects in an early phase, thereby preserving or improving survivors’ long-term health.

Despite growing care needs, access to survivorship care remains unequal across Europe. While some countries provide comprehensive follow-up programs, others offer limited or no survivorship care. For this reason, the PanCareFollowUp (PCFU) Consortium was established in 2018 as part of a European Union Horizon 2020-funded project [5–7]. The consortium of 14 partners from ten European countries, launched the PCFU project to empower CAYA cancer survivors across Europe and improve their health and quality of life by promoting high-quality cancer survivorship care [5, 8]. As part of this initiative, an innovative survivorship care intervention building on the care model as established in the Netherlands was developed and evaluated: the PCFU Care intervention [6]. The PCFU Care intervention is grounded in two key elements of high-quality care: (1) evidence-based guidelines for surveillance of late effects [7, 9]; and (2) the concept of person-centred care (PCC) [10–13]. Clinical practice guidelines are widely regarded as powerful tools to improve the quality of care, reduce variability in daily practice, and reduce costs [9]. Accordingly, the PCFU Consortium formulated recommendations for surveillance of late effects of CAYA cancer [7].

PCC, the second key pillar of the PCFU Care intervention, has been incorporated into the definition of high-quality cancer survivorship care [14, 15]. PCC, defined as *‘care that is respectful of and responsive to individual patient preferences, needs, and values, and ensuring that patient values guide all clinical decisions’* [14], actively involves patients as partners and places the person’s perspective on their life situation and health condition at the centre of care. A collaborative partnership between patient and healthcare professionals (HCPs) in PCC empowers CAYA cancer survivors to take an active role in managing their health and behaviour [16]. Given the lifelong increased risk of late effects after cancer treatment, survivors must decide when to seek professional care and when to manage issues independently. Thus, supporting self-management and engagement is a key element of the PCFU Care intervention. Self-management requires knowledge, skills, and confidence, competencies which can vary between survivors [17]. The PCC approach focuses on enhancing survivors’ skills and resources by sharing knowledge, boosting confidence, and promoting readiness for prevention and intervention [18]. The core principles of PCC include: (1) respect for the survivors’ values, preferences and needs; (2) a holistic approach with psychosocial support and physical comfort to handle late effects; (3) information and communication to inform and engage the survivors in their follow-up care and (4) a caring culture with care coordination for continuity and transition. Underlying these principles is the process of shared decision-making, through which the most appropriate care decisions are reached in collaboration with the survivor [13, 19].

To support the implementation of PCC in the PCFU Care intervention, and to ensure a consistent approach across participating healthcare settings, a training package was developed outlining key PCC principles and their practical application in cancer survivorship care. The training package comprises two key components: (1) a training presentation with voice-over introducing the concept of PCC; and (2) a workshop focused on sustainable implementation of PCC in the context of cancer survivorship care delivery. Because PCC requires changes in roles, workflows, and service organization, the guide was informed by three complementary frameworks (Table 1): (1) At the survivor-HCP consultation level, Ekman’s three pillars of PCC — *initiation*, *integration*, and *safeguarding* the partnership — provided the conceptual basis for building and maintaining survivor–provider partnership (Supplementary Fig. 1) [10]; (2) At the departmental level, the five drivers to excellence in PCC developed by Planetree International, highlight the importance of aligning values, organizational structures, clinical practices, and measurement systems to support PCC within clinical teams [20, 21]; (3) Across multiple levels (including survivor, HCP and healthcare system), the frameworks by Frampton and Santana emphasize that multi-level PCC implementation requires institutional endorsement, a strong commitment to change, and leadership with a vision aligned with PCC principles, in order to achieve sustainable adoption across organisations [21, 22] (Supplementary Fig. 2).

**Table 1:** Frameworks applied in the training package for PCC implementation in the PCFU Care Intervention^a^

Framework	Level	Core concepts	Application in PCFU Care Intervention
Ekman et al. - Three pillars [10]	Survivor-HCP level	1. Initiating the partnership	Consultation preparation: exchange of information between the HCP and the survivor.
		2. Integrating the partnership	Collaborative discussion and shared decision-making using the structured PCC consultation framework (Supplementary Fig. 1).
		3. Safeguarding the partnership	Use of a survivor care plan to safeguard and formalize the partnership.
Planetree - Five drivers [20, 21]	Department level	1. Creating organizational structures that promote engagement	Identification of organizational prerequisites for PCC implementation in survivorship care
		2. Connecting values, strategies and actions	Input for HCP training learning goals, including partnership, communication, and values-based practice
		3. Implementing practices that promote partnership	Translation into implementation strategies within participating clinical sites
		4. Knowing what matters	Incorporation of survivor perspectives into care planning
		5. Using evidence to drive improvement	Use of PCC evidence to guide care practices and continuous improvement
Frampton & Santana - PCC implementation (Supplementary Fig. 2) [20, 22]	Multi-level (survivor, HCP, healthcare system)	1. Structure	• Requirements for the healthcare system/organisation
		2. Process	• Cultivating communication • Respectful and compassionate care • Engaging survivors in managing their care • Integration of care
		3. Outcomes	• Practice-related outputs • Engagement-related outcomes • Access to care

^a^The core concepts from Ekman and Planetree were integrated throughout the training package (presentation and workshop) as overarching principles, rather than being addressed as discrete components. The framework by Frampton and Santana primarily informed the broader structural and organisational conditions required for PCC implementation. The training package mainly focussed on the process components of this framework.

This paper describes the evaluation of experiences of HCPs and supporting staff with the training package for the implementation of PCC in the PCFU Care intervention.

## Methods

### Training package of implementation of PCC in the PCFU Care intervention

The PCFU Care intervention integrated PCC within a prospective cohort study including 800 CAYA cancer survivors conducted across four European clinics in Belgium, Czech Republic, Italy and Sweden [8]. All centres provided long-term follow-up care, either within paediatric oncology settings (Belgium and Italy) or adult oncology centres (Czech Republic and Sweden). To support and strengthen the integration of PCC within the intervention, a training package was developed in close collaboration with survivors from Childhood Cancer International Europe, who were actively involved in both the design and conduct of the guide. This training package comprised two key components: (1) a training presentation with voice-over on the introduction of PCC; and (2) a four- hour workshop on sustainable implementation of PCC in the context of cancer survivorship care delivery. Both the training presentation and the workshop were supported by a reference book on PCC, which included an introduction to PCC, an outline of the workshop, printable consultation cards for HCPs and additional relevant literature. A detailed overview of the learning outcomes, educational methods, and content of both the training presentation and the workshop is provided in Supplementary Table 1.

#### Training presentation with voice-over

A narrated training presentation, lasting approximately one hour, was developed for the HCPs (i.e. physicians, nurses, and support staff) involved in daily survivorship care at one of the four clinics participating in the PCFU Care intervention. It was delivered as an asynchronous self-paced module and made available to participants via Dropbox as a downloadable Powerpoint file. To ensure consistency in delivery of the PCFU Care intervention, this material was distributed to the HCPs of the four participating study sites prior to the enrolment of the first survivor in the intervention study. Due to COVID-19 restrictions, in-person workshops at the clinics were not feasible, so the training presentation served as the primary means of introducing the essential concepts of PCC. In the presentation, the three pillars of PCC by Ekman [10] and the five drivers for the implementation of PCC as developed by Planetree International were introduced [20, 21]. The training presentation also underscored that PCC implementation is an ongoing process of development and improvement, shaped by input from both survivors and the healthcare team.

#### Workshop on PCC based on the PCFU adjusted conceptual framework

Six months after the enrolment of the first survivor in the prospective cohort study, a workshop on PCC was organized at the four study sites of the project, as COVID-19-related restrictions prevented earlier implementation. The workshop was structured around the conceptual framework, with emphasis on key PCC process domains, including communication, engagement, and integration of care. The primary aim of the workshop was to provide HCPs with a clear framework and practical tools to obtain a sustainable implementation of PCC in their daily practice. The workshop lasted approximately four hours and was facilitated by a PCC expert with extensive experience as the head of a cancer survivorship care clinic that had been awarded a Planetree International Golden Certificate for excellence in PCC. The other facilitator had been trained by the PCC expert. Both facilitators were prepared using a standardized protocol to ensure consistency across sites. The workshop was divided into five sections: (1) an introduction and session on expectations, providing an open forum for participants to express their feelings, knowledge, and potential resistance to PCC; (2) a summary of principles of PCC in relation to the project; (3) exploration of PCC from the perspective of healthcare providers; (4) exploration of PCC from the perspective of survivors; and (5) an evaluation and planning session to determine next steps for implementation at each centre. The workshop was tailored through interactive discussions in which participants identified their prior experience with PCC, initial resistance or obstacles, and - at the end of the session- remaining barriers and preferred strategies to address them, allowing the content to be adapted to each group’s needs. While informal feedback was shared during discussions, only systematically collected data sources were used for evaluation and reporting.

### Application frameworks in the PCC training package

Based on the PCC implementation frameworks, two consultation‑supporting tools were developed and introduced as part of the training presentation and workshop: (1) a seven‑step PCC consultation structure (Supplementary Fig. 2) and (2) a shared decision‑making visual aid (Supplementary Fig. 3). In addition, the frameworks informed the learning outcomes of the training presentation and workshop, which aimed to support HCPs in recognizing the core principles of PCC, applying these principles in daily survivorship care practice, identifying physician and nurse perspectives that influence survivor care management, and identifying survivor perspectives that shape care needs. A key intended outcome was to support HCPs in integrating survivor perspectives into care planning and follow-up, thereby strengthening survivor–HCP partnerships in line with PCC principles.

#### Evaluation of the training presentation and workshop

Both the training presentation and workshop were evaluated using study-specific questionnaires at the four participating PCFU Care clinics. Participants in the evaluation included HCPs and (research) support staff involved in implementing the PCFU Care intervention. Questionnaires were completed immediately after attending the training presentation and/or workshop. The questionnaires were developed by the study team in a structured manner based on the goals of the PCC training package, focusing on whether these goals were achieved and on participants’ experiences with the training. A follow-up evaluation of the workshop was originally planned but could not be conducted because COVID-19–related delays in workshop delivery left insufficient time within the project period.

The *training presentation* questionnaire assessed participants’ familiarity with person-centred care (PCC), the usefulness of the presentation and reference book, and the impact on four key domains: (1) readiness (preparedness to implement PCC); (2) importance (perceived relevance of PCC); (3) confidence (self-assessed ability to apply PCC before and after the training presentation); and (4) knowledge perceived improvement in understanding PCC). The questionnaire concluded with items on the helpfulness of presentation elements and open-ended questions about barriers and facilitators, missing content, and suggestions for improvement. The *workshop* questionnaire focused on perceived quality (usefulness for professional activities, overall impression, and most/least valuable aspects), relevance (fulfilment of educational goals), suitability of the format (e.g. time for discussion), and anticipated impact on clinical practice regarding PCC. It concluded with an open-ended question about how the workshop might influence future practice. The full questionnaires are provided in the supplementary material (Supplementary Material S1, Supplementary Material S2).

## Results

### Evaluation of the training presentation with voice-over

Eighteen participants (mostly female physicians, median age 49) completed the training presentation questionnaire (Table 2). All clinics were represented.

**Table 2:** Demographic characteristics participants PCC training presentation (*n* = 18) and PCC workshop (*n* = 28)

	Training presentation participants *n* = 18 *n*(%)	Workshop participants *n* = 28 *n*(%)
Female sex	14 (77.8%)	12 (66.6%)
Age, years^a^	49 (18.8)	-^b^
Country		
Italy	5 (27.8%)	8 (27.8%)
Sweden	2 (11.1%)	3 (11.1%)
Belgium	5 (27.8%)	8 (27.8%)
Czech Republic	6 (33.3%)	9 (33.3%)
Profession		
MD (paediatrician, paediatric (haemato-) oncologist, (research) doctor)	11 (61.1%)	11 (39.3%)
Nurse	3 (16.7%)	4 (14.3%)
Allied health professionals (psychologist, nutritionist)	2 (11.1%)	7 (25%)
Research supporting professions (research coordinator, research manager, research secretary)	2 (11.1%)	6 (21.4%)

^a^ Median (IQR)

^b^ Age of the participants of the workshop unknown

**Fig. 1:**
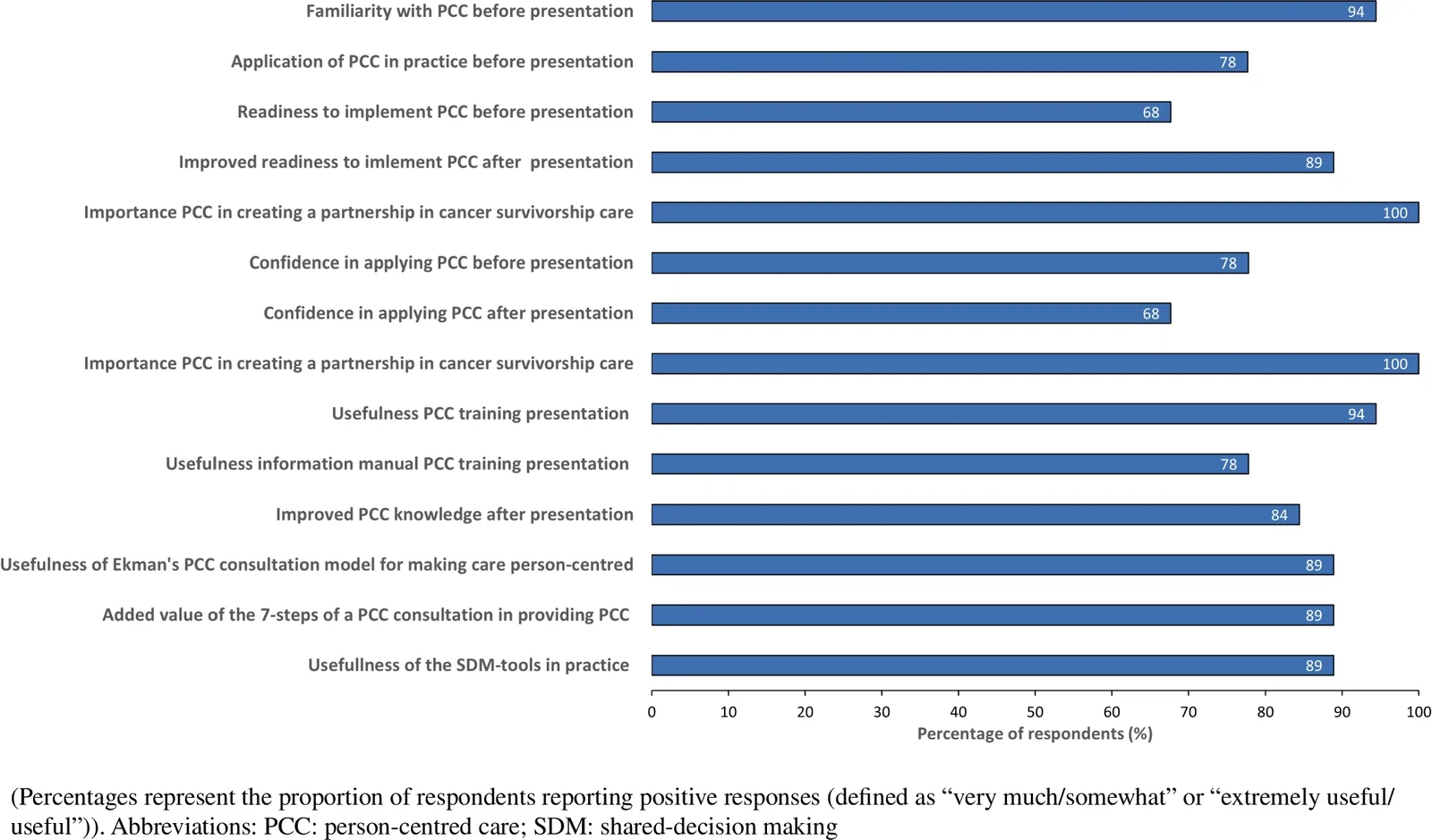
Percentage of HCPs reporting positive outcomes following the PCC training presentation (*n* = 18)

Prior to the training presentation, almost all (94%) participants were (somewhat or very much) familiar with the concept of PCC (Fig. 1). In total, 78% already applied PCC (i.e. as establishing a partnership with the patient) in daily clinical practice in some way. Readiness to implement PCC rose from 68% before the presentation to 89% afterwards. Confidence in applying PCC declined from 78% pre-presentation to 68% post-presentation. All participants acknowledged the importance of PCC and the presentation was rated useful by almost all participants (94%). The information manual was less valued (78% positive respondents). Knowledge improved, with 94% of respondents reporting at least some increase. Ekman’s consultation model, the seven steps of a PCC consultation and the SDM-tools were perceived as useful by almost 9/10 respondents.

Reported barriers included limited consultation time, staff shortages, and weak digital infrastructure. To support implementation, they proposed more time, collaboration, multilingual resources, and training. They valued collective empowerment and holistic care but asked for more practical examples and survivor insights. While the materials were seen as useful and aligned with current practice, participants emphasized the need for clearer, actionable guidance to better integrate PCC into routine care.

### Evaluation of workshop

Of the 32 workshop attendees, 28 completed the questionnaire. Two-thirds were female, and all countries except Sweden were equally represented (8–9 participants each). Medical doctors formed the largest group (39%), followed by allied health professionals (25%) (Table 2).

**Fig. 2:**
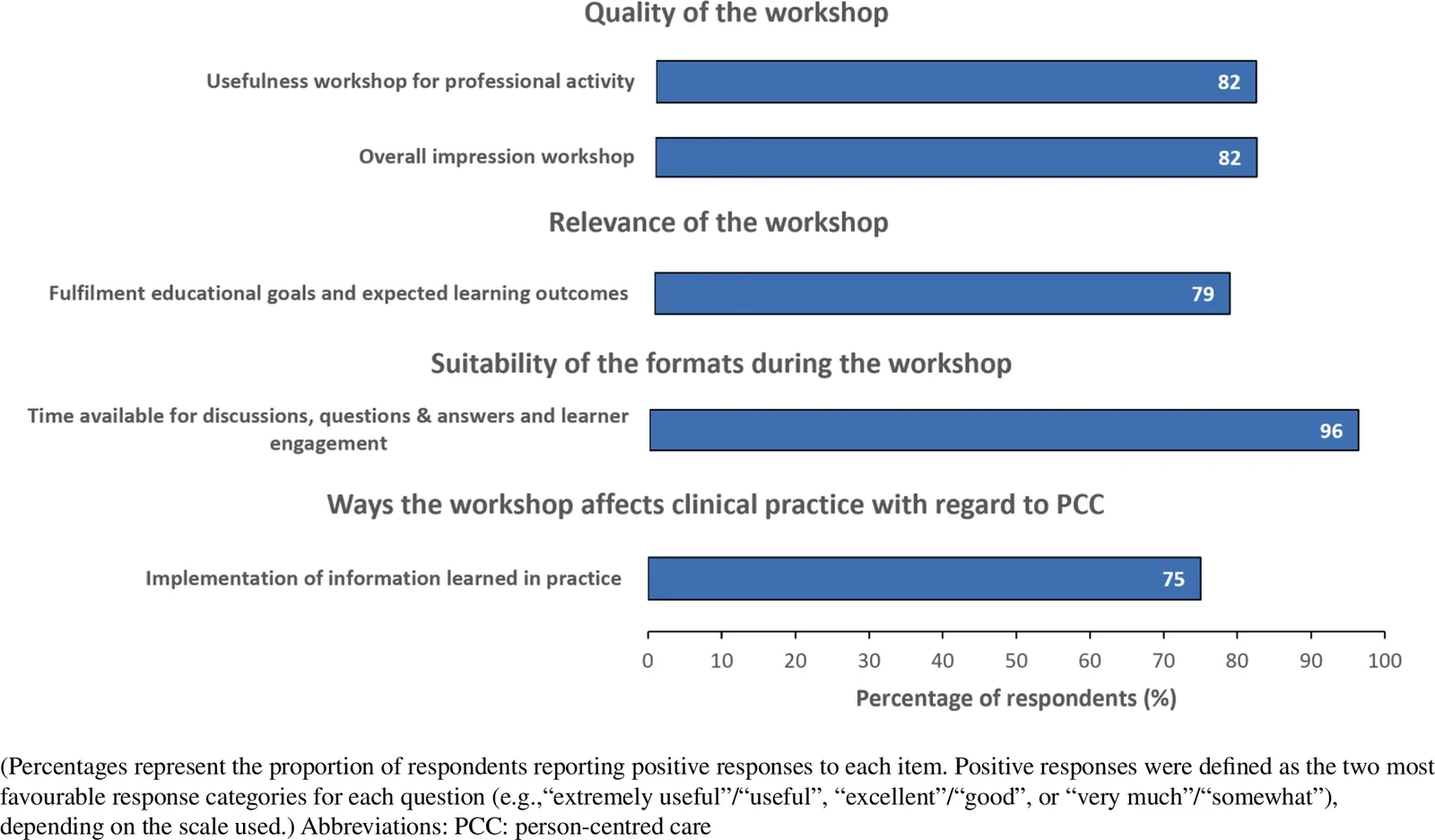
Percentage of healthcare professionals reporting positive outcomes following the PCC workshop (*n* = 28)

Participants rated the workshop positively: 82% found it useful for their professional activities, and 82% reported a positive overall impression (Fig. 2). Regarding relevance, 79% indicated that the workshop met their educational goals. The workshop was well received, with 96% confirming sufficient time for discussion. Expectations regarding implementation in practice were more varied, with 75% indicating they would likely apply the content in their clinical work. Participants valued open dialogue, peer exchange, and international perspectives, especially comparisons with Dutch practice and practical examples. Other strengths included the multidisciplinary setup, survivor input, and focus on system-level improvements. Suggested improvements included extending the workshop duration. Participants planned to apply insights by enhancing empathy, listening, team collaboration, and shared decision-making. Some intended to initiate team meetings, improve care coordination, and provide better information on late effects. Reported barriers included administrative, technological, and resource constraints. Still, the workshop was seen as inspirational and valuable for further reflection and discussion.

## Discussion

This study evaluated a two component training package for implementing PCC within the PCFU Care intervention, comprising a voice-over a training presentation and a four-hour interactive workshop with additional information. While both components were positively received, the findings also highlight several practical considerations relevant to the implementation and sustainability of PCC in survivorship care. Our findings suggest that PCC is broadly recognized as essential within survivorship care. The training with voice-over supported clear improvements in participants’ perceived readiness and knowledge. Moreover, indicating that even relatively concise educational interventions can have meaningful educational impact. These results are consistent with earlier research by Dwamena et al. showing that even short training interventions can successfully transfer person-centred skills to HCPs [23]. In line with other studies, this underscores the role of training, alongside organizational factors and leadership, as an important facilitator of PCC implementation [32]. Together, these findings underscore the value of targeted educational initiatives as a foundational element of PCC implementation strategies. Feedback from participants provided important insights into the practical challenges of implementing PCC in clinical practice. The observed decline in confidence following the training presentation, together with the challenges discussed during the workshop, suggest that acquiring knowledge alone may not be sufficient to support confident application of PCC in daily practice. Reported challenges related to time constraints, staffing, availability of practical examples, and infrastructure highlight the importance of aligning training content with everyday clinical realities. Based on participant feedback, future iterations of the training package will place greater emphasis on applied learning approaches, such as case-based examples and practice-oriented tools, alongside strategies that address organizational barriers. Such refinements may help facilitate the translation of PCC principles into routine survivorship care.

A key lesson from this project relates to the logistical feasibility of implementing PCC training in busy clinical settings. The combination of an asynchronous training presentation and a four-hour interactive workshop was deliberately chosen, as delivering a full in-person workshop at the start of the intervention was not feasible due to COVID-19 restrictions. Repeated exposure to PCC principles was also considered beneficial for learning and reinforcement. While the training presentation enabled HCPs to engage with PCC concepts at their own pace, participation in the workshop required dedicated time, which proved challenging for some participating centres. These experiences highlight the importance of flexible delivery formats and institutional support when implementing PCC interventions in routine practice. Future offerings may benefit from modular or blended approaches that further minimize disruption to clinical workflows. Where logistical constraints limit participation, the training presentation may serve as an accessible initial introduction to PCC principles; however, when feasible, an in-person workshop appears important to support deeper skill development and practical application in daily clinical practice. These findings also highlight the importance of effective facilitation. In particular, facilitators should not only have expertise in PCC but also be able to adapt discussions to the local clinical context and actively engage participants in interactive dialogue, as this was highly valued during the workshops.

This study also reinforces that successful PCC implementation depends not only on individual competencies but also on structural and organizational conditions. Multilevel involvement, at the level of HCPs, institutions, and healthcare systems, is therefore essential to foster sustainable cultural change towards PCC [24]. The implementation of PCC in the context of the PCFU project might be different from implementation of PCC in a single institute as the PCFU consortium played an important role in the conduction of pre-requisites for PCC. These pre-requisites such as the survivor questionnaire and a template for the survivor care plan have been disseminated in a replication manual and are accessible for all clinics in Europe. Besides the efforts of the PCFU consortium, participating institutes are important for creating the organizational structure for a PCC culture, leadership and commitment to change, supporting a workforce committed to PCC, providing a PCC environment and developing and integrating structures to support health information technology [22, 25].

Key strengths of this study include the dual-component training package, with the training presentation being a first encounter with PCC and an interactive live workshop allowing participants to ask questions directly to the PCC expert. The workshop was tailored to the specific context of each clinic, ensuring personalized support. Both the voice-over training presentation and the workshop were evaluated by many participants, providing valuable feedback. However, due to delay in the project resulting from the COVID-19 restrictions, it was not feasible to conduct follow-up assessments after PCC implementation to evaluate longer-term changes in knowledge, confidence, or practice. Additionally, it is unclear whether all participants regularly see cancer survivors in their practice.

In conclusion, this study presents both the opportunities and challenges of implementing PCC within survivorship care. Training interventions enhanced HCPs’ readiness and knowledge, and PCC frameworks were well received. At the same time, barriers such as time constraints, staffing limitations, and digital infrastructure must be addressed to achieve sustainable implementation. Embedding PCC in cancer survivorship care, as promoted by the PCFU project, has the potential to enhance accessibility, enable earlier detection and intervention for late effects, and ultimately improve quality of life for CAYA cancer survivors. Sustainable implementation, however, requires careful attention to training design, delivery formats, and organizational context, as well as opportunities for applied learning. For organizations seeking to adopt similar interventions, key considerations include combining flexible training formats with interactive components, ensuring institutional support for protected training time, and integrating practical, context-specific learning tools. By addressing these factors, the PCFU Care training package provides actionable insights to support broader adoption of PCC approaches within survivorship care settings.

## Supplementary Information

Below is the link to the electronic supplementary material.Supplementary file1 (DOCX 463 KB)Supplementary file2 (DOCX 188 KB)Supplementary file3 (DOCX 26.0 KB)Supplementary file4 (DOCX 394 KB)Supplementary file5 (DOCX 18.6 KB)Supplementary file6 (DOCX 20.7 KB)

## Data Availability

The data that support the findings of this study are available from the corresponding author upon reasonable request.
