# Supplementary material for: A multi‑country evaluation of healthcare professionals’ experiences with the PanCareFollowUp training package to implement person-centred care in childhood cancer survivorship care: A PanCareFollowUp study
Source: Support Care Cancer. 2026 Jun 30;34(7):703. doi: 10.1007/s00520-026-10930-5 (PMC13314809; doi:10.1007/s00520-026-10930-5)
Supplement: Supplementary file 2 [file 520_2026_10930_MOESM2_ESM.docx]

**Supplementary Figure 2.** Framework for PCC implementation in the PCFU Care intervention – adapted from Frampton and Santana [1, 2]^a^

^a^ Note: This figure provides a conceptual overview of the multi-level organisational conditions (structure), care interactions (process), and outcomes required for PCC implementation. It complements Table 1, which focuses on the practical application of theoretical frameworks within the PCFU Care intervention and training package. The training package primarily focused on the process components of this framework, while the structural conditions required for PCC implementation were addressed at the institutional level and supported by the PCFU Consortium. 

**References**

1.	Frampton, S., et al., *Harnessing Evidence and Experience to Change Culture: A Guiding Framework for Patient and Family Engaged Care.* NAM Perspectives, 2017. **7**.

2.	Santana, M.J., et al., *How to practice person-centred care: A conceptual framework.* Health Expect, 2018. **21**(2): p. 429-440.
