# Supplementary material for: A multi‑country evaluation of healthcare professionals’ experiences with the PanCareFollowUp training package to implement person-centred care in childhood cancer survivorship care: A PanCareFollowUp study
Source: Support Care Cancer. 2026 Jun 30;34(7):703. doi: 10.1007/s00520-026-10930-5 (PMC13314809; doi:10.1007/s00520-026-10930-5)
Supplement: Supplementary file 3 [file 520_2026_10930_MOESM3_ESM.docx]

**Supplementary Table** **1****.** Overview of the PCC Training Presentation and Workshop within the PCFU Care Intervention

|  | Training presentation with voice-over | Workshop |
| --- | --- | --- |
| Purpose | To introduce HCPs to the principles of PCC and its relevance to survivorship care within the PCFU Care intervention. | To support sustainable implementation of PCC in daily clinical practice by translating PCC principles into practical skills and organizational action. |
| Target audience | Physicians, nurses, and allied healthcare professionals involved in daily cancer survivorship care at participating study sites | Physicians, nurses, and allied healthcare professionals involved in daily cancer survivorship care at participating study sites |
| Format | Asynchronous, self‑paced Microsoft PowerPoint presentation with embedded voice‑over. | Interactive, face‑to‑face workshop |
| Delivery/ facilitation | Distributed via Dropbox as a downloadable file with embedded voice-over | Facilitated by PCC experts from the PCFU project team using a standardized workshop structure across sites |
| Learning goals | To recognize the principles of person-centred survivorship care. To understand the relevance of PCC for daily survivorship care practice To reflect on self-reported readiness to apply PCC concepts | To recognize  the principles of person-centred survivorship care. To apply the principles of person-centred care in daily practice. To identify physician and nurse perspectives that influence survivor care management. To identify survivor perspectives that shape survivor care management. To integrate the survivor perspectives in survivor care. |
| Educational methods | Asynchronous, didactic instruction delivered through narrated slides and supported by self-directed learning. | Group discussions, listening exercises, reflective discussions informed by survivor-generated statements, and collaborative planning activities |
| Theoretical frameworks | Introduction to the three pillars of PCC [1], Planetree drivers [2, 3] and framework by Frampton and Santana [4] | Introduction to the three pillars of PCC [1], Planetree drivers [2, 3] and framework by Frampton and Santana [4] |
| Sections training presentation/workshop | 1. Principles of Person-Centred Survivorship Care 2. Person-Centred Survivorship Care from a Healthcare Professional Perspective 3. Person-centred Survivorship Care from a Survivor Perspective | 1. Welcome & personal introduction and expectation management of participants 2. Summary of Principles of Person-Centred Survivorship Care in relation to the project 3. Person-Centred Survivorship Care from a Healthcare Professional Perspective Identifying physician and nurse perspectives and values that influence survivor care management Further integration of Person-Centred Care principles in the context of survivorship 4. Person-Centred Survivorship Care from a Survivor Perspective Identifying survivor perspectives that shape survivorship care management Further integration of survivor perspectives into daily survivorship care 5. Reflections on Person-Centred Survivorship Care Group reflections on workshop topics Take home assignments and messages |
| Time investment | ±1 hour | ±4 hours |
| Evaluation | Post-training questionnaire | Post-workshop questionnaire, anecdotal feedback from participants |

* References

1.	Ekman, I., et al., *Person-centred care--ready for prime time.* Eur J Cardiovasc Nurs, 2011. **10**(4): p. 248-51.

2.	Frampton, S., et al., *Harnessing Evidence and Experience to Change Culture: A Guiding Framework for Patient and Family Engaged Care.* NAM Perspectives, 2017. **7**.

3.	Guastello, S. and K. Jay, *Improving the patient experience through a comprehensive performance framework to evaluate excellence in person-centred care.* BMJ Open Qual, 2019. **8**(4): p. e000737.

4.	Santana, M.J., et al., *How to practice person-centred care: A conceptual framework.* Health Expect, 2018. **21**(2): p. 429-440.
