# Supplementary material for: A multi‑country evaluation of healthcare professionals’ experiences with the PanCareFollowUp training package to implement person-centred care in childhood cancer survivorship care: A PanCareFollowUp study
Source: Support Care Cancer. 2026 Jun 30;34(7):703. doi: 10.1007/s00520-026-10930-5 (PMC13314809; doi:10.1007/s00520-026-10930-5)
Supplement: Supplementary file 4 [file 520_2026_10930_MOESM4_ESM.docx]

**Supplementary Figure 2.** Prompts to support shared decision making
