# Supplementary material for: A multi‑country evaluation of healthcare professionals’ experiences with the PanCareFollowUp training package to implement person-centred care in childhood cancer survivorship care: A PanCareFollowUp study
Source: Support Care Cancer. 2026 Jun 30;34(7):703. doi: 10.1007/s00520-026-10930-5 (PMC13314809; doi:10.1007/s00520-026-10930-5)
Supplement: Supplementary file 5 [file 520_2026_10930_MOESM5_ESM.docx]

**Evaluation of the PCC training presentation**

*One of the tasks of the PanCareFollowUp project is to organize a workshop to introduce the principles of Person Centred Care (PCC) to healthcare providers (HCPs) and to introduce strategies on how to integrate PCC principles into daily care practice. Due to the Covid pandemic the workshop was replaced by a presentation for introduction to PCC. With this questionnaire we would like to evaluate the presentation and to adjust the future activities to introduce PCC accordingly.* 

Gender: ……………………………………………………………………………………

Age: ………………………………………………………………………………………….

Profession: ……………………………………………………………………………….

Date of assessment: …………………………………………………………………

Country: ………………………………………………………………….

1. Were you familiar with Person Centred Survivorship Care (PCSC), as to create partnership with your patient, before you attended the presentation?

Very much---somewhat---not much---not at all---undecided

2. Did you apply Person Centred Care, as to create partnership with your patient, in your daily practice, before the presentation?

Very much---somewhat---not much---not at all---undecided

3. Did you attend the meeting with the presentation of Person Centred Care?   

Yes/no

4. Did you read the information manual with the goals of the presentation? 

Yes/no

5. How useful did you find the presentation of Person Centred Care for your daily clinical practice in the PanCareFollowUp project?

Extremely useful---useful---fairly useful--- not useful

6. How useful did you find the information manual for your daily clinical practice in the PanCareFollowUp project?

Extremely useful---useful---fairly useful--- not useful---not applicable

*Readiness*

7. Were you ready to implement PCC in cancer survivorship care, before the presentation?

Very much---somewhat---not much---not at all---undecided

8. Did the presentation improve your readiness for implementation of PCC?

Very much---somewhat---not much---not at all---undecided

*Importance:*

9. How important do you think Person Centred Care to create partnership is for cancer survivorship care?

Very much---somewhat---not much---not at all---undecided

*Confidence* 

10. Did you apply Person Centred Survivorship Care in the consultations *with confidence* before the presentation?

Very much---somewhat---not much---not at all---undecided

11. Do you apply Person Centred Survivorship Care in the consultations *with more confidence* after the presentation?

Very much---somewhat---not much---not at all---undecided

*Knowledge:* 

12. After attending the presentation, was your knowledge on how to apply Person Centred Care to create partnership, improved?

Very much---somewhat---not much---not at all---undecided

13. Is the organization of care according to the three steps of partnership, particularly ‘initiating, integrating and safeguarding’, helpful for making care person centred?

Very much---somewhat---not much---not at all---undecided

14. Are the 7 steps to a PCSC consultation an addition to your consultation practice to Person Centred Survivorship Care (PCSC)?

Very much---somewhat---not much---not at all---undecided

15. How helpful are the three questions for Shared Decision Making for the use in the clinical practice of shared decision making?

Very much---somewhat---not much---not at all---undecided

16. What are possible obstructions/pitfalls in implementing PCSC in your practice?

………………………………………………………………………………………………………………………… 

17. What would be helpful for you to implement PCSC successfully?

………………………………………………………………………………………………………………………….

18. What part of the information of the presentation or manual was most useful in your daily practice of PCC?

…………………………………………………………………………………………………………………………..

19. What information of the presentation or manual was least useful?

………………………………………………………………………………………………………………………….

20. What additional information was missing /would have been useful for implementing PCC in your daily practice?

…………………………………………………………………………………………………………………………..

21. Do you have any suggestions for improving the presentation?

………………………………………………………………………………………………………………………….

22. Do you have any suggestions for improving the Information manual?

………………………………………………………………………………………………………………………….
