# Supplementary material for: A multi‑country evaluation of healthcare professionals’ experiences with the PanCareFollowUp training package to implement person-centred care in childhood cancer survivorship care: A PanCareFollowUp study
Source: Support Care Cancer. 2026 Jun 30;34(7):703. doi: 10.1007/s00520-026-10930-5 (PMC13314809; doi:10.1007/s00520-026-10930-5)
Supplement: Supplementary file 6 [file 520_2026_10930_MOESM6_ESM.docx]

1) **Quality of the event (Implementation of Person Centered Care)**

How useful for your professional activity did you find this event?

| **Extremely useful** | **Useful** | **Fairly useful** | **Not useful** |
| --- | --- | --- | --- |
|  |  |  |  |

If this activity was not useful, please explain why: …………………………………………………………………………………

What was your overall impression of this event?

| **Excellent** | **Good** | **Fairly good** | **Poor** | **Very poor** |
| --- | --- | --- | --- | --- |
|  |  |  |  |  |

What was the best aspect of this event? ……………………………………………………………………………………………….

What was the worst aspect of this event? ……………………………………………………………………………………………..

2) **Relevance of the event (Implementation of Person Centered Care)**

Did the event fulfil your educational goals and expected learning outcomes?

| **Very much** | **Somewhat** | **Not much** | **Not at all** | **Undecided** |
| --- | --- | --- | --- | --- |
|  |  |  |  |  |

Please explain: ……………………………………………………………………………………………….

3) **Suitability of formats used during the event**

Was there adequate time available for discussions, questions & answers and learner engagement?

| **Yes,** **always/almost** **always** | **Yes, sometimes** | **Only rarely** | **Never** | **Undecided** |
| --- | --- | --- | --- | --- |
|  |  |  |  |  |

4) **Ways the event affects clinical practice with regard to Person Centered Care**

Will the information you learnt be implemented in your practice?

| **Very much** | **Somewhat** | **Not much** | **Not at all** | **Undecided** |
| --- | --- | --- | --- | --- |
|  |  |  |  |  |

Please explain:…………………………………………………………………….

Can you provide ONE example how this event will influence your future practice?

………………………………………………………………………………………
